# Autoencoder-Enhanced Convolutional Neural Networks for Plantar Pressure–Based Gait Pattern Recognition: Model Development and Cross-Validated Evaluation Study

**DOI:** 10.2196/88488

**Published:** 2026-04-21

**Authors:** Chuan-Chun Chang, Chi-Wen Lung, Yih-Kuen Jan, Qi-Qian Lu, Yi-You Wang, Yi-Sheng Chen, Ben-Yi Liau

**Affiliations:** 1 Department of Automatic Control Engineering Feng Chia University Taichung Taiwan; 2 Department of Creative Product Design Asia University Taichung Taiwan; 3 Rehabilitation Engineering Lab Department of Health and Kinesiology University of Illinois Urbana-Champaign Urbana, IL United States; 4 Department of Communications Engineering Feng Chia University Taichung Taiwan

**Keywords:** plantar pressure, gait, convolutional neural networks, autoencoder, wearable sensors, deep learning, machine learning, biomedical engineering

## Abstract

**Background:**

Plantar pressure imaging is a stable modality that reflects gait-related biomechanical characteristics and has been used increasingly for gait assessment and recognition. However, plantar pressure images are high dimensional and nonlinear, making manual feature engineering and conventional machine learning insufficient to capture discriminative patterns.

**Objective:**

This study aimed to develop a gait pattern recognition model based on plantar pressure using an autoencoder (AE)-enhanced convolutional neural network (CNN) and to evaluate its performance against baseline deep learning and classical machine learning approaches.

**Methods:**

A total of 13 healthy volunteers (aged 18-24 years) were recruited. Plantar pressure data were collected during treadmill walking using an in-shoe pressure measurement system and converted into frame-wise plantar pressure images. We compared a lightweight CNN (Light CNN), an AE-CNN cascade model, and an encoder-augmented CNN with an additional bottleneck layer. Model development used participant-wise data partitioning, and performance was evaluated using accuracy, precision, recall, and *F*_1_-score.

**Results:**

The proposed encoder-augmented CNN achieved the best overall performance (*F*_1_-score=96.20%), outperforming the Light CNN (*F*_1_-score=94.44%) and AE-CNN cascade (*F*_1_-score=92.45%). Confusion matrices and learning curves further indicated stable training behavior and consistent classification performance across gait patterns.

**Conclusions:**

Integrating representation learning (AE-based compression) with CNN-based classification improved the recognition of gait patterns from plantar pressure images. This pilot study included only healthy participants. Future work should validate generalizability in larger and clinically diverse cohorts and further investigate participant-level evaluation and model interpretability, as well as deployment feasibility.

## Introduction

With the rapid development of smart health care and artificial intelligence, the analysis of physiological signals has become increasingly important in fields such as disease diagnosis, rehabilitation monitoring, and identity verification. Among these signals, plantar pressure imaging is a stable method that reflects the posture of the individual, the characteristics of the gait, and foot functionality. Due to its ease of collection and visualization, plantar pressure imaging has been widely used for behavioral analysis, clinical evaluation, and biometric recognition. Gait is a fundamental human activity that represents the coordinated movement of the lower limbs. Although often overlooked because of its automaticity, gait involves complex and synchronized interactions between the musculoskeletal system and neural control. Understanding gait dynamics is important to assess gait alterations and support rehabilitation planning. Previous studies have investigated gait analysis using deep spatiotemporal learning and machine learning, including pathological gait classification, gait phase detection, pilot gait classification studies, and deep learning–based gait trajectory modeling [1-4]. Typically, the gait cycle can be described by the stance and swing phases, and more detailed phase characterization may provide additional functional information [2,4].

Traditional gait and plantar pressure analysis relied on manual feature extraction and classical machine learning algorithms. However, plantar pressure data are typically high dimensional and nonlinear, making it difficult for handcrafted features to capture informative patterns consistently. In recent years, deep learning, particularly convolutional neural networks (CNNs), has shown superior performance in automatically extracting features and improving classification accuracy. Autoencoder (AE) techniques have also been used to reduce dimensionality and alleviate redundancy, improving representation compactness and computational efficiency for high-dimensional inputs. Previous research has explored plantar pressure–based assessment in health-related applications. Deschamps et al [5] analyzed plantar pressure distribution patterns in people with diabetes, and Amemiya et al [6] investigated the relationships between elevated plantar pressure and gait characteristics in patients with diabetes. Wang et al [7] developed an insole-based gait monitoring technique to recognize gait patterns associated with knee osteoarthritis. These studies highlight the value of plantar pressure analysis to quantify lower limb loading characteristics and support gait-related assessment. Other researchers have investigated computational models for gait recognition. Nguyen et al [8] used smart shoes to classify ambulatory activities and proposed statistical characteristics combined with conventional classifiers, and Jeong et al [9] studied the classification of activity with respect to stairs using plantar pressure sensors. Jun et al [10] further proposed a hybrid deep learning framework that integrates plantar pressure images and 3D skeletal data, indicating that multimodal fusion can improve the recognition of abnormal gait patterns compared to pressure-only inputs. Beyond classification, related gait evaluation studies have compared optimization strategies for sensory data classification using deep neural networks [11] and evaluated machine learning algorithms for electromyography pattern classification in gait disorders [12], underscoring the broader demand for robust learning pipelines. To address the need for portable, real-time applications, Cho [13] developed a deep learning approach using plantar pressure signals to estimate walking speed and gait-related classification tasks. Chhoeum et al [14] applied CNN-based regression to estimate knee joint angles using foot pressure mapping images. Ling et al [15] introduced an AE-CNN–based multisource data fusion framework to estimate the step length of the gait motion, illustrating the practical value of representation learning when handling high-dimensional gait-related data. Ardhianto et al [16] formulated the estimation of the foot progression angle as an object detection task on plantar pressure images using YOLO (You Only Look Once)-based models, demonstrating the feasibility of computer vision–style pipelines on pressure maps. From a system-level perspective, Zhou et al [17] developed a gait detection and plantar pressure analysis system using a flexible triboelectric pressure sensor array and deep learning, highlighting the direction of continuous wearable gait monitoring under real-world constraints. Collectively, these studies suggest that plantar pressure imaging is advantageous due to its stability, visualization quality, and strong correspondence with gait cycles. Building on these advances, there remains a need for pressure-only deep learning frameworks that can learn discriminative representations from high-dimensional plantar pressure images, reduce redundancy via representation learning (eg, AEs), and maintain computational efficiency. Importantly, clinical claims should be supported by validation in clinically diverse cohorts; therefore, model development and evaluation in controlled settings should be clearly distinguished from future clinical verification.

## Methods

### Study Design and Workflow

**Figure 1:**
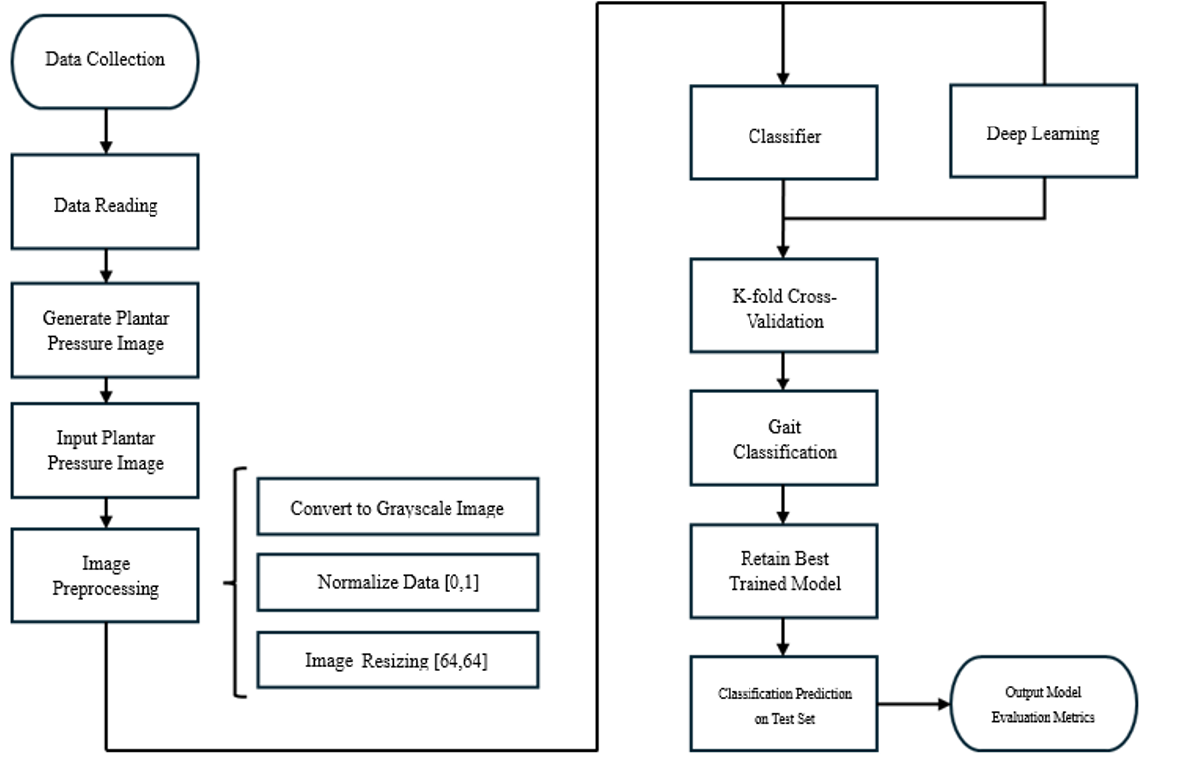
Overall workflow of the proposed plantar pressure–based recognition system.

The general workflow of this study is illustrated in Figure 1. The experimental design consisted of 3 major components. The first component involved the collection of plantar pressure response data required to generate plantar pressure images. The second component focused on the application of machine learning classifiers and a deep learning model based on a CNN. The third component addressed the AE, which was further investigated as a core part of this study. In the final stage of the experiments, the CNN and AE frameworks were integrated to perform classification and performance evaluation.

**Figure 2:**
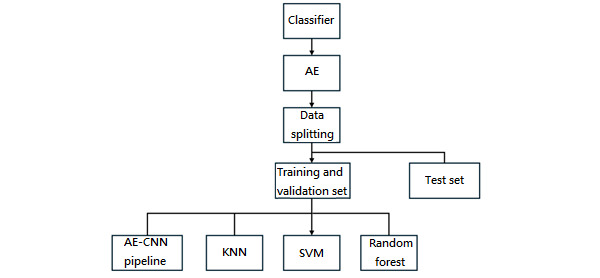
Model development framework that includes the autoencoder (AE)–convolutional neural network (CNN) pipeline and traditional classifiers. AE: autoencoder; AE-CNN: autoencoder convolutional neural network; KNN: k-nearest neighbors; SVM: support vector machine.

**Figure 3:**
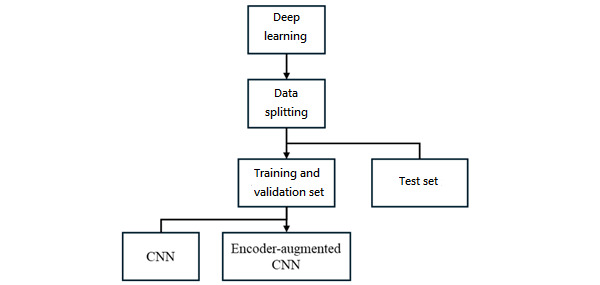
Architectures of the convolutional neural network (CNN) and encoder-augmented CNN models.

As shown in Figure 1, the experimental workflow highlights the sequential architecture of the study. In particular, the second component, which encompasses both classifiers and CNN-based models, is presented in separate blocks. The detailed structures of these blocks are illustrated in Figures 2 and 3.

In the classifier block diagram, the AE-CNN cascade is defined as an AE in which compressed data are modularized and sequentially integrated with a CNN. This design enables the model to be trained in a staged manner, thereby forming the AE-CNN cascade. Furthermore, because its data processing pathway resembles that of conventional classifiers, the AE-CNN cascade was grouped within the classifier block for consistency in experimental design.

In the deep learning block diagram, the encoder-augmented CNN is defined as a hybrid model in which the encoder component of the AE is directly integrated into the CNN classifier. This design enables end-to-end training, allowing the encoder and CNN to fuse into a unified framework. By adopting this hybrid strategy, the model is trained as an integrated architecture, which is referred to as encoder-augmented CNN.

### Participants and Experimental Setup

A total of 13 healthy university student volunteers (aged 18-24 years) were recruited for this study. Exclusion criteria included current or previous foot ulcers, diabetes, vascular disease, hypertension, inability to walk independently for at least 10 minutes, and continued use of medications that could affect gait. Only participants who met all eligibility criteria were enrolled.

### Ethical Considerations

This study was approved by the Central Regional Research Ethics Committee of China Medical University, Taichung, Taiwan (approval CRREC-112-130). All participants received a full explanation of the study procedures and provided their written informed consent prior to participation. The data were deidentified for analysis and reporting. No financial compensation was provided to participants in this study.

### Plantar Pressure Measurement

**Figure 4:**
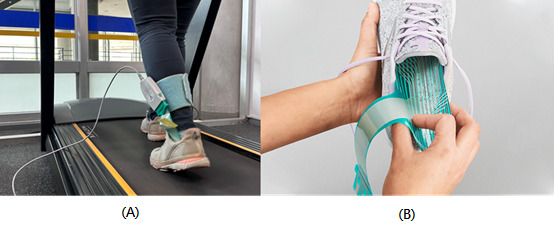
F-Scan plantar pressure sensor: (A) data collection wearing mode and (B) pressure sensing sheet [18].

Plantar pressure data were acquired using the Tekscan F-Scan in-shoe pressure measurement system (Figure 4 [18]). The F-Scan provides high-resolution plantar pressure distribution with real-time acquisition via a dedicated data cable, making it suitable for gait analysis, sports science, insole design, and clinical applications. The key functions adopted in this study are summarized in Table 1. In particular, the system’s real-time recording capability and high spatial resolution enabled the capture of subtle pressure changes, thereby improving the fidelity of the experimental dataset.

**Table 1:** Main functions of the F-Scan plantar pressure sensor.

Item	Explanation	Application
Plantar pressure measurement	Unit: kPa	Measurement of plantar pressure distribution
Gait analysis	Analysis of step length, step frequency, and gait cycle	Walking, running, and abnormal gait
High-resolution sensor	0.5 cm² per sensing unit; visualization of subtle pressure variations	Detailed plantar pressure analysis
Calibration function	Three calibration methods: walking, gait, and point modes	Normal gait analysis, abnormal gait analysis, and plantar pressure distribution while standing

### Foot Pressure Data Acquisition

Plantar pressure responses were recorded using the Tekscan F-Scan in-shoe system while participants walked on a treadmill at a fixed speed. Each trial began with a 1-minute familiarization period to stabilize gait at the target speed. The F-Scan microsensors sampled plantar pressure at 25 Hz, and instantaneous pressures were stored as matrix-valued frames and exported in CSV format for downstream processing.

**Figure 5:**
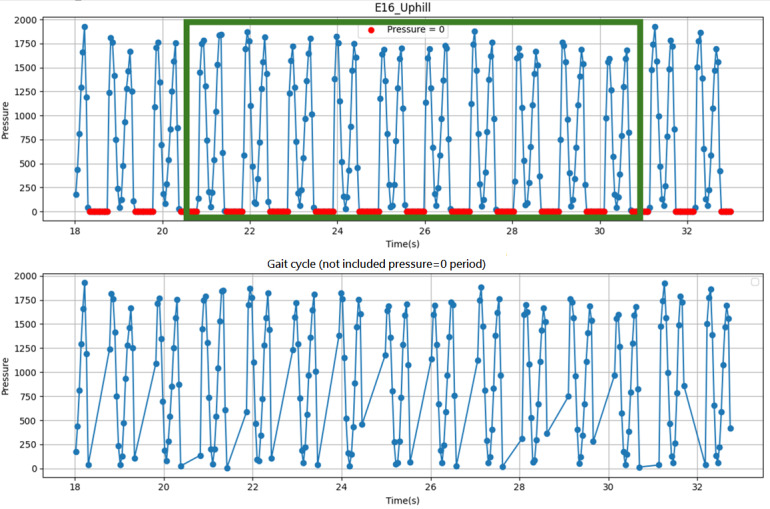
Example of the plantar pressure response across gait cycles: the green bracket indicates 10 gait cycles, blue markers denote stance-phase frames, and red markers denote swing-phase frames.

Frame selection was based on gait cycles, each consisting of a stance phase and a swing phase (Figure 5). The pressure value at each time point was calculated as the sum of pressure readings across all sensing elements (kPa) as an overall loading indicator. In Figure 5, the green bracket denotes 10 gait cycles, blue markers indicate frames within the stance phase, and red markers denote the swing phase. As the plantar load is negligible during swing, the sensors yield near-zero readings. Therefore, to enable frame-wise classification of gait patterns and only the stance-phase frames of the 10 cycles were retained for analysis.

The final dataset comprised 6994 frames in 3 gait patterns: slow walking (2590 frames), fast walking (2162 frames), and uphill walking (2242 frames). These frames served as input to the proposed models for training and evaluation.

### Image Preprocessing

**Figure 6:**
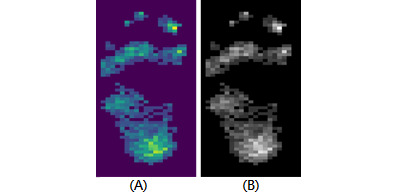
(A) Example of an image of plantar pressure distribution rendered with a perceptually uniform viridis colormap. (B) The corresponding grayscale image used as the model input after preprocessing.

Matrix-form plantar pressure signals exported by the F-Scan system were converted into frame-wise instantaneous plantar pressure matrices using Python 3.10.12 (Python Software Foundation) to enable deep learning. Each frame-wise matrix was normalized to the range (0, 1) using min-max normalization and rendered directly as a grayscale intensity image, in which pixel intensity represents normalized pressure magnitude. For visualization (Figure 6A), we additionally rendered the same normalized matrices as pseudocolor pressure maps using the perceptually uniform viridis colormap. The viridis colormap was used for visualization only. The grayscale images were resized to 64×64 pixels to ensure consistency across samples, and Gaussian noise was injected as a lightweight augmentation to improve noise robustness and mitigate overfitting. After preprocessing, the resulting input tensor for model training and evaluation had the shape (6994, 64, 64, 1).

### Model Architecture

In this study, 3 deep learning architectures were developed to address plantar pressure image classification, each designed with distinct structural characteristics. The first was a lightweight CNN (Light CNN), which served as the baseline model. It was constructed with 3 convolutional layers, each followed by max-pooling to progressively reduce dimensionality while preserving salient spatial features. Batch normalization was incorporated to stabilize the training process, while dropout layers were used to mitigate overfitting. A fully connected layer and a final softmax classifier were used to output the probabilities of the 3 gait categories, namely, slow walking, fast walking, and uphill walking. The structural design of this network is summarized in Table 2, which establishes the baseline framework for subsequent comparisons.

**Table 2:** Light convolutional neural network model architecture.

Tasks	Structural parameters		Training parameters (activation function)
	Layer	Detailed parameters	
Feature extraction	Input image	(64, 64, 1)	—^a^
Feature extraction	Conv2D_1	32,(3, 3), padding=“same”; L2 regularization (λ=0.0005)	Leaky ReLU
Feature extraction	MaxPool2D	(2, 2), padding=“same”	—
Feature extraction	Conv2D_2	64,(3, 3), padding=“same”; L2 regularization (λ=0.0005)	Leaky ReLU
Feature extraction	MaxPool2D	(2, 2), padding=“same”	—
Feature extraction	Conv2D_3	128,(3, 3), padding=“same”; L2 regularization (λ=0.0005)	Leaky ReLU
Feature extraction	MaxPool2D	(2, 2), padding=“same”	—
Feature extraction	Dropout (0.2)	—	—
Multiclass classification	Dense	90	—
Multiclass classification	Dense	60	—
Multiclass classification	Dense	3	Softmax
Other parameters	K-fold	9	—
Other parameters	Batch size	64	—
Other parameters	Epochs	100	—
Other parameters	Loss function	categorical_crossentropy	—
Other parameters	Optimizer	Adam (learning rate=0.0005)	—
Other parameters	Metrics	accuracy	—

^a^Not applicable.

The second model was the AE-CNN cascade, which modularized the integration of an AE and a CNN classifier. In this design, the encoder of the AE first compressed the high-dimensional plantar pressure matrices into a compact latent representation, while the decoder simultaneously ensured that essential structural information could be reconstructed. The compressed latent features were then transferred to a CNN classifier for further convolutional processing and final classification. This staged cascade design effectively separated feature compression from classification, improving both the stability and the interpretability of the learning process. The encoder architecture used in the AE-CNN cascade is presented in Table 3, illustrating how feature reduction and classification were sequentially integrated.

**Table 3:** Autoencoder encoder model architecture.

Task	Structural parameters		Training parameters (activation function)
	Layer	Detailed parameters	
Encoded	Input image	(64, 64, 1)	—^a^
Encoded	Conv2D_1	32, (3, 3), padding=“same”	Leaky ReLU
Encoded	MaxPool2D	(2, 2), padding=“same”	—
Encoded	Conv2D_2	64, (3, 3), padding=“same”	Leaky ReLU
Encoded	MaxPool2D	(2, 2), padding=“same”	—
Encoded	Conv2D_3	128, (3,3), padding=“same”	Leaky ReLU
Encoded	MaxPool2D	(2, 2), padding=“same”	—
Encoded	Flatten	—	—
Encoded	Dense	128	—

^a^Not applicable.

To evaluate generalization while reducing the risk of information leakage from correlated frame-wise samples, data partitioning was performed at the participant level. Participants (n=13) were first split into a development set (n=10, 76.9%) and an independent held-out test set (n=3, 23.1%). Within the development set, a participant-wise grouped 9-fold cross-validation procedure was applied for model selection and stability assessment. In each fold, all frame-wise samples from participants in the training folds were used for model fitting, and all samples from participants in the held-out fold were used for validation. As 10 is not evenly divisible by 9, fold sizes differed by at most 1 participant (8 folds contained 1 participant and 1 fold contained 2 participants). After cross-validation, the final model configuration was retrained on the full development set and evaluated once on the held-out test set. Performance was reported using accuracy, precision, recall, and *F*_1_-score.

The third architecture was the encoder-augmented CNN, which further extended the integration of AE and CNN by embedding the encoder directly into the CNN pipeline. Unlike the cascade structure, this hybrid design adopted an end-to-end framework, where the encoder served as the initial feature extractor and its outputs were directly connected to the subsequent CNN layers. This approach allowed the encoder and CNN to be jointly optimized, combining compact representation learning with the discriminative power of deep convolutional layers. The architectural layout of this model is summarized in Table 4, highlighting its streamlined structure and enhanced learning efficiency.

**Table 4:** Encoder-augmented convolutional neural network model architecture.

Task	Structural parameters				Training parameters (activation function)
	Layer	Detailed parameters			
Spatial and feature compression	Input image	(64, 64, 1)			—^a^
Spatial and feature compression	Conv2D_1	32,(3, 3), padding=“same”; L2 regularization (λ=0.0005)			—
Spatial and feature compression	Batch normalization	—			Leaky ReLU
Spatial and feature compression	MaxPool2D	(2, 2), padding=“same”			—
Spatial and feature compression	Conv2D_2	64,(3, 3), padding=“same”; L2 regularization (λ=0.0005)			Leaky ReLU
Spatial and feature compression	MaxPool2D	(2, 2), padding=“same”			—
Spatial and feature compression	Conv2D_3	128,(3, 3), padding=“same”; L2 regularization (λ=0.0005)			Leaky ReLU
Spatial and feature compression	MaxPool2D	(2, 2), padding=“same”			—
Spatial and feature compression	Dropout(0.2)	—			—
Spatial and feature compression	Flatten	—			—
Spatial and feature compression	Dense (bottleneck)	128			—
Multiclass classification	Dense	90			—
Multiclass classification	Dense	60			—
Multiclass classification	Dense	3			Softmax
Other parameters	K-fold,		9	—	
Other parameters	Batch size		64	—	
Other parameters	Epochs		100	—	
Other parameters	Loss function		categorical_crossentropy	—	
Other parameters	Optimizer		Adam (learning rate=0.0005)	—	
Other parameters	Metrics		accuracy	—	

^a^Not applicable.

### Evaluation Metrics

Model performance was assessed using accuracy, precision, recall (sensitivity), *F*_1_-score, and confusion matrix:

- The accuracy reflects the overall proportion of correctly classified samples in all classes.
- Precision quantifies the reliability of positive predictions for a given class by indicating how many predicted positives are correct.
- Recall (sensitivity) measures the model’s ability to correctly identify true instances of a given class.
- The *F*_1_-score provides a balanced summary of precision and recall, particularly useful when both false positives and false negatives matter.
- The confusion matrix offers a class-by-class view of predictions vs ground truth, enabling identification of error patterns (eg, which gait classes are most frequently confused). For the 3-class setting in this study (slow walking, fast walking, and uphill walking), a 3×3 matrix was used to summarize the results per class and general trends.

All metrics were computed on the designated evaluation split without using any evaluation data for parameter updating. Unless stated otherwise, results are reported at the overall level to facilitate comparison among the baseline CNN, the AE-CNN cascade, the encoder-augmented CNN, and traditional classifiers; confusion matrices are additionally provided to illustrate class-wise error patterns. To reduce optimistic bias due to correlated frame-wise samples, all data splitting was performed at the participant level. Specifically, all stance-phase frames from the same participant were assigned to a single fold during cross-validation, ensuring that no participant’s frames appeared in both the training and validation sets within any iteration. Model performance was summarized using accuracy, precision, recall, and *F*_1_-score.

### Reporting Guidelines

This model development and evaluation study was reported with reference to the CREMLS (Consolidated Reporting of Machine Learning Studies) checklist. The completed checklist is provided in Multimedia Appendix 1.

## Results

### Overview

This section summarizes the main comparative findings from the deep learning and classical machine learning models evaluated. Detailed training curves, optimization experiments, and hyperparameter tuning results are provided in Multimedia Appendix 2.

Among the baseline deep learning models, the Light CNN achieved an *F*_1_-score of 94.44% on the held-out test set, whereas the AE-CNN cascade achieved an *F*_1_-score of 92.45%.

Additional optimization analyses of the encoder-augmented CNN, including comparisons of downsampling strategies, batch normalization configurations, and bottleneck layer inclusion, supported the final model design reported here (Multimedia Appendix 2).

For the classical machine learning models trained on AE-derived features, support vector machine (SVM) with a radial basis function kernel performed best (*F*_1_-score=93.76%), followed by k-nearest neighbors (*F*_1_-score=91.73%) and random forest (*F*_1_-score=88.54%).

### Principal Findings

**Figure 7:**
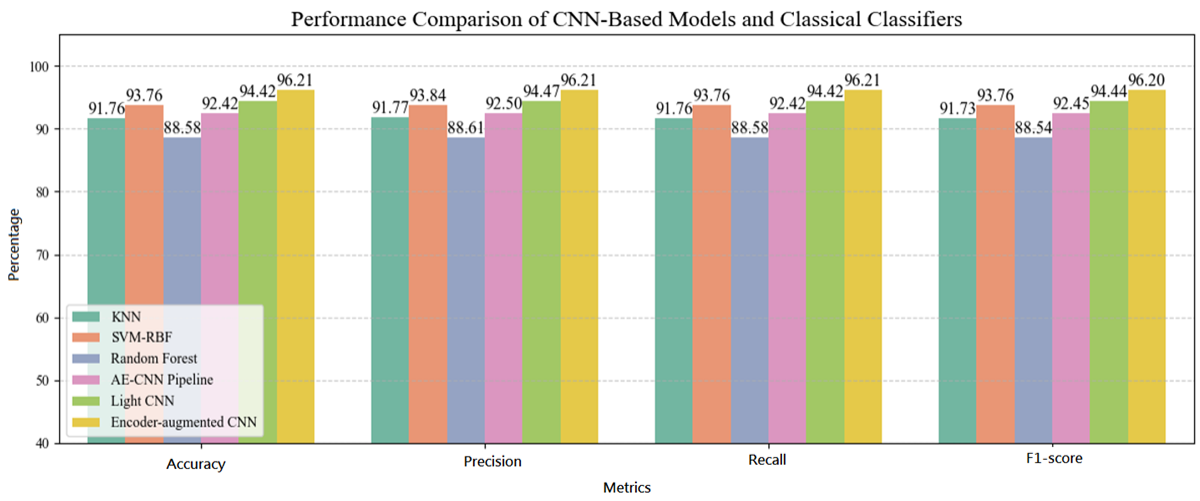
Overall performance comparison of all models. AE-CNN: autoencoder convolutional neural network; KNN: k-nearest neighbors; SVM-RBF: support vector machine-radial basis function.

The primary finding of this study is that the proposed encoder-augmented CNN architecture achieves superior performance (*F*_1_-score=96.20%) in classifying dynamic gait patterns from plantar pressure images compared to both baseline deep learning models and classical machine learning classifiers (Figure 7; Table 5). This result supported the hypothesis that an integrated deep learning architecture, combining the feature extraction and dimensionality reduction capabilities of an AE with the classification power of a CNN, is highly effective for this task.

**Table 5:** Overall performance in the held-out test set.

Model	Accuracy	Precision	Recall	*F*_1_-score
KNN^a^	91.76	91.77	91.76	91.73
SVM^b^-RBF^c^	93.76	93.84	93.76	93.76
Random forest	88.58	88.61	88.58	88.54
AE^d^-CNN^e^ cascade	92.42	92.50	92.42	92.45
Light CNN	94.42	94.47	94.42	94.44
Encoder-augmented CNN	96.21	96.21	96.21	96.20

^a^KNN: k-nearest neighbors.

^b^SVM: support vector machine.

^c^RBF: radial basis function.

^d^AE: autoencoder.

^e^CNN: convolutional neural network.

## Discussion

### Overview

This model development and evaluation study demonstrates that the integration of AE-based representation learning with CNN architectures can achieve accurate recognition of gait patterns based on plantar pressure in a pilot dataset of healthy participants. Future work should validate generalization across broader demographics and clinical populations, evaluate robustness to real-world variability (eg, footwear, sensor noise, and speed fluctuations), and further strengthen interpretability and deployment feasibility for wearable or embedded applications. The performance of the Light CNN (*F*_1_-score=94.44%) also demonstrated that even a lightweight, stand-alone CNN can effectively learn discriminative features from plantar pressure data. Among the classical methods, the **support vector machine**–RBF classifier proved to be the most robust, outperforming **k-nearest neighbors** and random forest, which aligns with its known strengths in handling high-dimensional feature spaces.

The study also highlights the importance of architectural choices in model optimization. Supplementary analyses of downsampling strategy, batch normalization placement, and bottleneck layer inclusion are provided in Multimedia Appendix 2. These experiments supported the final selection of the encoder-augmented CNN configuration reported in the main manuscript.

### Interpretation of Results and Gait Feature Analysis

**Figure 8:**
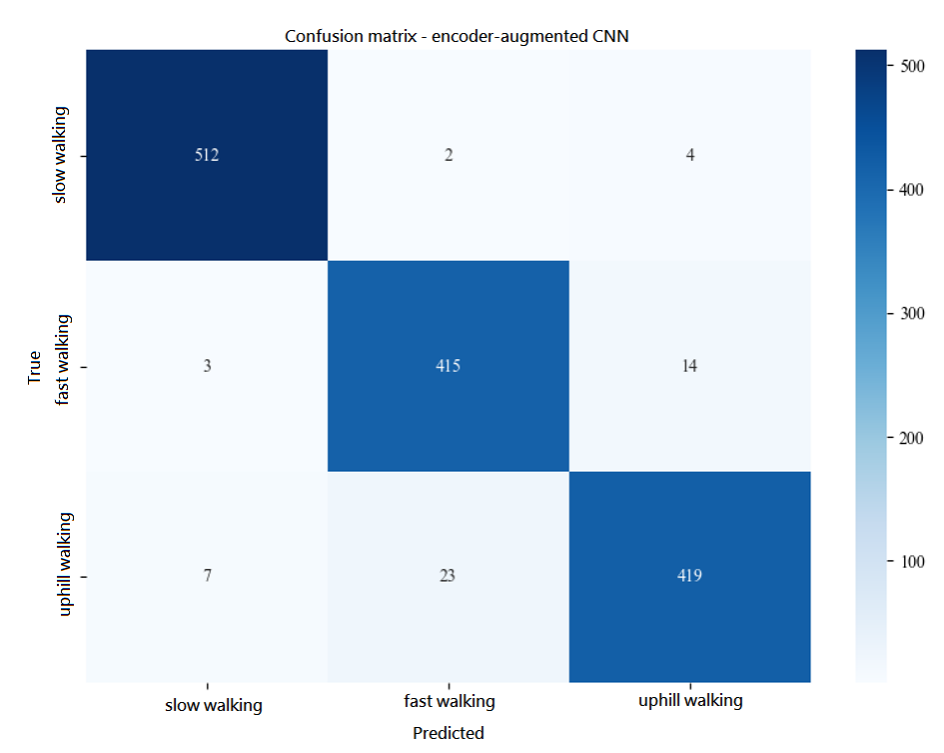
Confusion matrix for the encoder-augmented convolutional neural network (CNN).

A notable and consistent finding in multiple models was the confusion between “fast walking” and “uphill walking” gaits (Figure 8). The encoder-augmented CNN, for example, misclassified 23 “uphill” instances as “fast walking.” This suggests that at similar walking speeds, the plantar pressure distributions for these 2 activities share substantial similarities. The primary differentiator may lie in subtle temporal features or pressure shifts related to gravitational resistance during uphill walking, which current spatial feature–focused models may not fully capture. In contrast, “slow walking” was classified with very high precision, indicating that variations in walking speed produce more distinct plantar pressure patterns than variations in surface uphill.

### Comparison With Prior Work

The use of deep learning, particularly CNNs, for plantar pressure analysis is consistent with recent trends in biomechanics and clinical research. Although many studies have successfully used CNNs for static pressure images (eg, for disease diagnosis), this research extends their application to dynamic gait classification. The accuracy achieved by the encoder-augmented CNN (96.21%) is competitive with or exceeds that reported in other studies using different sensor modalities or classification algorithms for similar tasks. The finding that an integrated AE-CNN architecture outperforms a standard CNN suggests that explicit feature learning and dimensionality reduction prior to classification can be a beneficial strategy for complex, high-variance data such as plantar pressure sequences. Clinical and translational implications should be interpreted with caution. This pilot study included only healthy young adults under controlled treadmill conditions and did not include participants with pathological gaits (eg, diabetes-related gait alteration). Therefore, while plantar pressure imaging is clinically relevant and the proposed framework shows technical promise, clinical screening performance has not been empirically validated here and requires future studies in clinical cohorts and real-world environments. In addition, our current approach performs frame-wise classification of stance-phase plantar pressure maps and does not explicitly model temporal dynamics across gait cycles; future work could incorporate sequence models (eg, temporal CNNs or long short-term memory) to better capture temporal gait signatures.

### Limitations

Although this study has yielded promising results, several limitations should be considered:

1. Single data source—plantar pressure data in this study were collected from a limited number of participants in a controlled laboratory environment. Whether the model’s generalization ability can be extended to populations with different ages, genders, weights, or specific pathological gaits (eg, flat feet and diabetic foot) requires further validation.

2. Model interpretability—compared to classical machine learning models, deep learning models are often regarded as “black boxes,” with less transparent decision-making processes. Although this study validated the effectiveness of the model, it did not explore which specific regions or features of plantar pressure images the model used for classification.

3. Limited gait types—the study only covered 3 specific dynamic gaits. The applicability of the model to more complex daily activities, such as walking downhill, turning, or climbing stairs, has yet to be determined.

In addition, the evaluation in this study was reported primarily at the frame level. Although frame-wise metrics are useful for model comparison, participant-level performance (eg, aggregating frame-level predictions to participant-level decisions) should be reported in future studies to better reflect real-world use. Moreover, statistical uncertainty (eg, CIs via bootstrapping) and systematic robustness tests under controlled perturbations (eg, varying noise intensity or sensor shift) were not performed in this study and remain important directions for future work.

### Conclusions

This study developed and evaluated a deep learning architecture named encoder-augmented CNN for gait classification using plantar pressure images. The model combines the feature extraction capabilities of an AE with the classification strengths of a CNN. Through systematic structural optimization, it ultimately achieved an accuracy of 96.21% in the 3-class dynamic-gait recognition task, outperforming classical machine learning methods and other deep learning variants in this study.

On the basis of the findings and limitations of this research, future studies could proceed in the following directions:

- Database expansion and model generalization—recruit a more diverse range of participants and collect data in settings closer to real-life scenarios to validate and enhance the model’s generalization ability. Future clinical studies could explore whether this framework can assist in the assessment of pathological gaits.
- Enhancing the interpretability of the model—introduce visualization techniques (eg, gradient-weighted class activation mapping) to analyze the plantar pressure heat maps that the model focuses on during classification decisions. This would improve understanding of the basis for the model’s decisions and could potentially lead to the discovery of new biomechanical indicators.
- Multimodal data fusion—to address the confusion between fast walking and uphill walking, future work could attempt to fuse data from other sensors (such as gyroscopes and accelerometers) to build a multimodal gait recognition system, with the aim of achieving higher classification accuracy.
- Model lightweighting and real-time application—explore techniques such as model pruning or knowledge distillation to further reduce the computational complexity of the encoder-augmented CNN. This would enable its deployment on wearable devices or embedded systems for real-time gait monitoring and feedback.

## Data Availability

The datasets generated or analyzed during this study are not publicly available due to privacy and ethical restrictions but are available from the corresponding author on reasonable request. Access to deidentified data may be subject to institutional approval and a data use agreement, where applicable. Requests for data access may be directed to the corresponding author at byliau@fcu.edu.tw.

## Appendix

Multimedia Appendix 1 CREMLS_checklist.

Multimedia Appendix 2 Supporting optimization analyses, training curves, and hyperparameter tuning results.

## Abbreviations

AE: autoencoder
CNN: convolutional neural network
CREMLS: Consolidated Reporting of Machine Learning Studies
