# Supplementary material for: Autoencoder-Enhanced Convolutional Neural Networks for Plantar Pressure–Based Gait Pattern Recognition: Model Development and Cross-Validated Evaluation Study
Source: JMIR Form Res. 2026 Apr 21;10:e88488. doi: 10.2196/88488 (PMC13146233; doi:10.2196/88488)
Supplement: Multimedia Appendix 1 [file formative_v10i1e88488_app1.docx]

Multimedia Appendix 1. Consolidated Reporting of Machine Learning Studies (CREMLS) Checklist

| Item number | Item | Location in manuscript or rationale |
| --- | --- | --- |
| Study details |  |  |
| 1.1 | The medical or clinical task of interest | Abstract (Background/Objectives); Introduction (Background). |
| 1.2 | The research question | Abstract (Objective); Introduction. |
| 1.3 | Current medical or clinical practice | Introduction. |
| 1.4 | The known predictors and confounders of what is being predicted | Introduction; Methods (Participants and Experimental Setup). |
| 1.5 | The overall study design | Methods (Design and Workflow). |
| 1.6 | The medical institutional settings | Methods (Participants and Experimental Setup; Foot Pressure Data Acquisition). |
| 1.7 | The target patient population | Methods (Participants and Experimental Setup). Study participants were healthy young adults; therefore, the target population in this pilot study was nonpatient adults rather than patients. |
| 1.8 | The intended use of the ML model | Abstract (Objective); Introduction; Conclusions. |
| 1.9 | Existing model performance benchmarks for this task | Introduction; Discussion (Comparison with prior work). |
| 1.10 | Ethical and other regulatory approvals obtained | Methods (Ethics Approval). |
| The data |  |  |
| 2.1 | Inclusion or exclusion criteria for the patient cohort | Methods (Participants and Experimental Setup). |
| 2.2 | Methods of data collection | Methods (Foot Pressure Data Acquisition; Image Preprocessing). |
| 2.3 | Bias introduced due to the method of data collection used | Discussion (Limitations: single data source, controlled laboratory environment, limited number of subjects). |
| 2.4 | Data characteristics | Methods (Foot Pressure Data Acquisition; Image Preprocessing). The composition is reported in Methods. |
| 2.5 | Methods of data transformation and preprocessing applied | Methods (Image Preprocessing; Model Architecture). |
| 2.6 | Known quality issues with the data | Discussion (Limitations). No major sensor-specific quality issue analysis was reported separately. |
| 2.7 | Sample size calculation | Not applicable to this pilot model-development study; a formal sample size calculation was not performed. |
| 2.8 | Data availability | Data Availability statement. |
| Methodology |  |  |
| 3.1 | Strategies for handling missing data | Not explicitly reported. No dedicated missing data handling procedure was described. |
| 3.2 | Strategies for addressing class imbalance | No explicit class rebalancing method was reported. Class frequencies were reasonably comparable across the three gait conditions (slow: 2590 frames; fast: 2162 frames; uphill: 2242 frames). |
| 3.3 | Strategies for reducing dimensionality of data | Methods (Model Architecture). Dimensionality reduction/compressed representation was achieved through the autoencoder encoder in AE-CNN and Encoder-augmented CNN pipelines. |
| 3.4 | Strategies for handling outliers | Not explicitly reported. No separate outlier-handling procedure was described. |
| 3.5 | Strategies for data augmentation | Methods (Image Preprocessing). A lightweight data augmentation strategy was used: Gaussian noise was injected into the input matrices before training. |
| 3.6 | Strategies for model pretraining | External pre-training was not used. The encoder component was trained using the study data set within the model development pipeline. |
| 3.7 | The rationale for selecting the ML algorithm | Methods (Model Architecture). |
| 3.8 | The Method of evaluating model performance during training | Methods (Model Architecture; Evaluation Metrics). Participant-level train/validation/test splitting was used to monitor model performance during model development. |
| 3.9 | The method used for hyperparameter tuning | Methods and Multimedia Appendix 2. Hyperparameter and architecture optimization procedures are summarized in the supplementary appendix. |
| 3.10 | Model’s output adjustments | Not explicitly reported beyond standard multiclass class prediction derived from model outputs. |
| Evaluation |  |  |
| 4.1 | Performance metrics used to evaluate the model | Methods (Evaluation Metrics); Results; Table 5; Figures 7-8. |
| 4.2 | The cost or consequence of errors | Introduction and Discussion (especially Interpretation of Results and Gait Feature Analysis). The manuscript discusses why misclassification could affect the interpretation of gait-condition differences and subsequent clinical translation. |
| 4.3 | The results of internal validation | Results (held test set performance, overall model comparison, and confusion matrix). |
| 4.4 | The final model hyperparameters | Methods (architecture tables) and Multimedia Appendix 2 (optimization details and final model settings). |
| 4.5 | Model evaluation on an external data set | Not applicable. No external validation data set was used; evaluation was limited to the internal division at the participant level. |
| 4.6 | Characteristics Relevant for detecting data shift and drift | Not applicable. Data shift/drift assessment was not performed because the model was not evaluated in deployment or on a separate external dataset. |
| Explainability & transparency |  |  |
| 5.1 | The most important features and how they relate to the outcomes | Partially addressed in Discussion (Interpretation of Results and Gait Feature Analysis). No formal characteristics of importance, saliency or Grad-CAM analysis was reported. |
| 5.2 | Plausibility of model outputs | Discussion. |
| 5.3 | Interpretation of a model's results by an end user | Discussion and Conclusions. Potential end use is described, but no formal end user usability evaluation was performed. |
