# Supplementary material for: Autoencoder-Enhanced Convolutional Neural Networks for Plantar Pressure–Based Gait Pattern Recognition: Model Development and Cross-Validated Evaluation Study
Source: JMIR Form Res. 2026 Apr 21;10:e88488. doi: 10.2196/88488 (PMC13146233; doi:10.2196/88488)
Supplement: Multimedia Appendix 2 [file formative_v10i1e88488_app2.docx]

Multimedia Appendix 2. Supplementary Figures and Tables

This appendix contains supporting optimization analyses, training curves, and hyperparameter tuning results moved from the main manuscript to comply with journal limits on figures and tables while preserving methodological transparency.

**Performance of Baseline Deep Learning Models**

**Light CNN**

The Light CNN model was trained for 100 epochs. Its learning process, characterized by rapid convergence in the initial stages, is shown in Supplementary Figure S1. The model achieved a final training accuracy of approximately 95% and a validation accuracy that stabilized between 93-94%. On the held-out test set, this model yielded an F1-score of 94.44%.


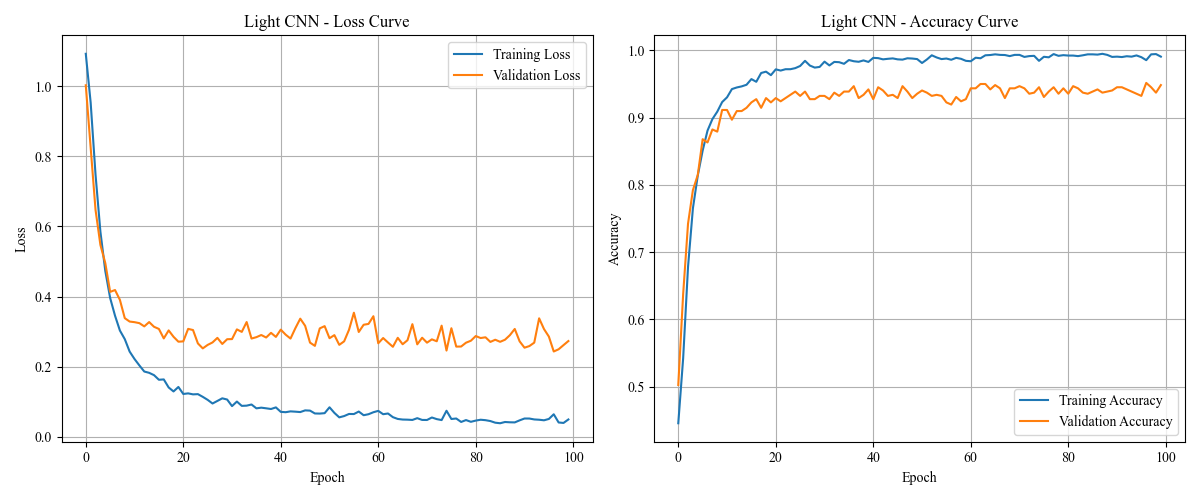


Supplementary Figure S1. Loss and Accuracy Curves for the Light CNN during Training.

**AE-CNN Cascade**

The AE-CNN Cascade model, which uses features extracted by an autoencoder, exhibited a more gradual learning curve compared to the Light CNN (Supplementary Figure S2). The training loss eventually stabilized below 0.2, while the validation loss settled between 0.35 and 0.40. The final training accuracy exceeded 92%, with a validation accuracy of about 90%. This model achieved an F1-score of 92.45% on the held-out test set.

**Impact of Downsampling Method**

Two downsampling methods were compared: convolution with a stride of 2 (stride = 2) and MaxPooling. Although their performance metrics were nearly identical (F1-scores of 94.66% for stride = 2 and 94.48% for MaxPooling), the training curves revealed that the stride = 2 model exhibited more pronounced oscillations in validation loss. Data analysis further supported this, showing a validation loss standard deviation of 0.1216 for the stride model, compared to a more stable 0.1027 for the MaxPooling model.

**Impact of Batch Normalization (BN)**

The effect of adding Batch Normalization (BN) layers was evaluated by testing configurations with BN placed after one, two, or three convolutional layers (1-BN, 2-BN, 3-BN). All three configurations improved performance during cross-validation, and the 3-BN configuration achieved the highest validation F1-score (96.57%; Supplementary Figure S3). However, increasing the number of BN layers also led to greater volatility in validation loss. Specifically, the 1-BN configuration had the lowest standard deviation of validation loss (0.1429), whereas the 3-BN configuration had the highest (0.2045) and required the longest training time (Supplementary Table S1). Considering the trade-off between performance, stability, and computational cost, we selected the 1-BN configuration for the final model reported on the held-out test set.

Supplementary Table S1. Training stability and computation time for BN configurations.

| Batch normalization | Validation set loss SD | Time taken (seconds) |
| --- | --- | --- |
| 1-BN | 0.1429 | 4856.49 |
| 2-BN | 0.1813 | 5613.07 |
| 3-BN | 0.2045 | 6032.17 |

**Impact of a Bottleneck Layer**

Finally, a bottleneck layer was incorporated into the optimized Encoder-augmented CNN architecture. This addition resulted in a slight performance boost across all metrics, increasing the F1-score from 95.72% to 96.20% (Supplementary Figure S4). The validation loss standard deviation remained nearly unchanged, and the training time increased only marginally.

**Performance of Classical Machine Learning Classifiers**

Using features extracted from the autoencoder, three classical classifiers were fine-tuned. For the K-Nearest Neighbors (KNN) algorithm, optimal performance was achieved at K=3 (Supplementary Figure S5). For the Support Vector Machine (SVM), the Radial Basis Function (RBF) kernel yielded the highest F1-score of 93.76% (Supplementary Figure S6). For the Random Forest, performance stabilized after approximately 30-35 trees, leading to the selection of 38 as the final parameter (Supplementary Figure S7).

A comparison of the optimized classifiers shows that SVM-RBF performed the best with an F1-score of 93.76%, followed by KNN with an F1-score of 91.73%. Random Forest had the lowest performance among the three, with an F1-score of 88.54% (Supplementary Figure S8).


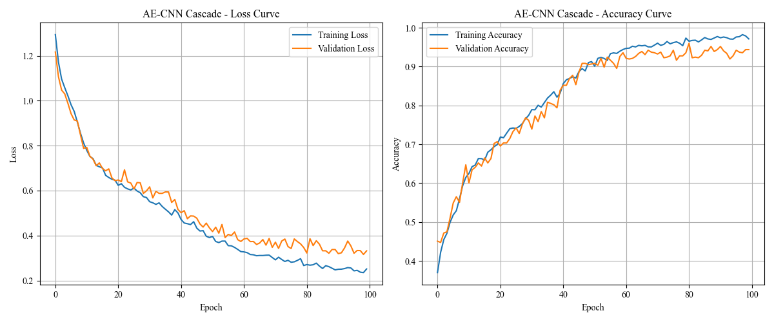


Supplementary Figure S2. Loss and Accuracy Curves for the AE-CNN Cascade during Training.


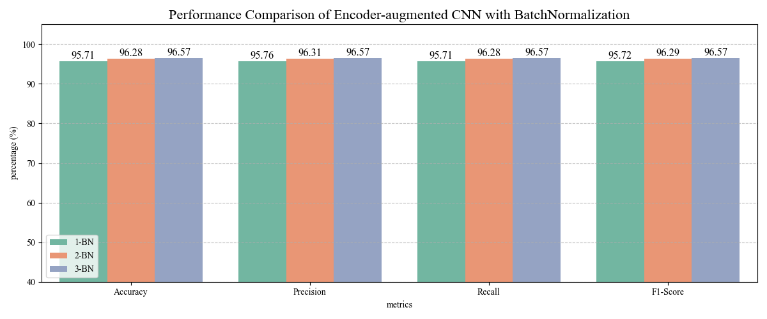


Supplementary Figure S3. Performance Comparison of the Encoder-augmented CNN with Different BN Configurations.


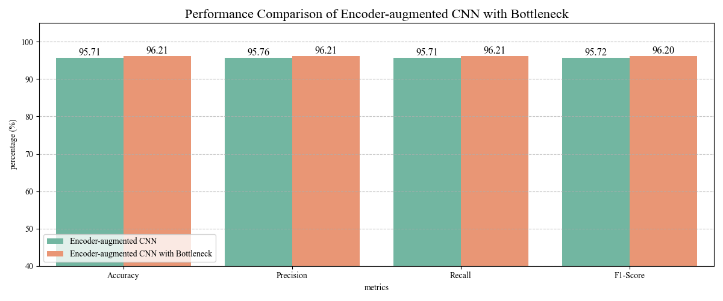


Supplementary Figure S4. Performance Comparison of the Encoder-augmented CNN With and Without a Bottleneck Layer.


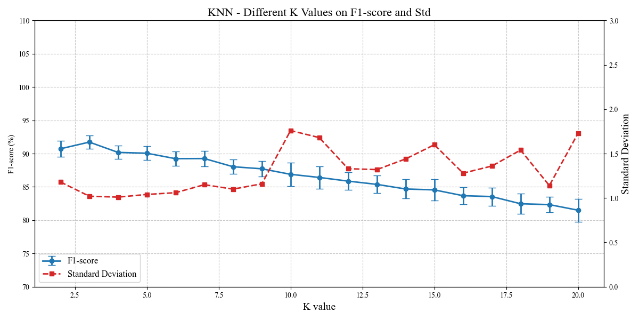


Supplementary Figure S5. Hyperparameter tuning process for the KNN classifier.


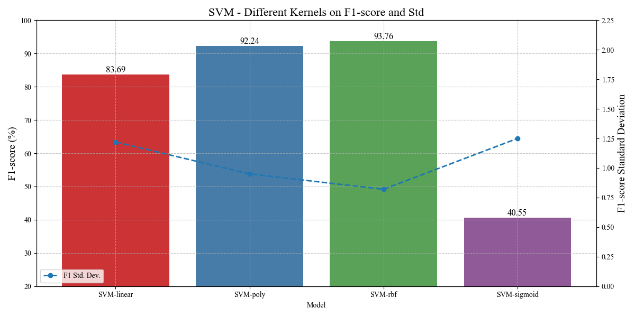


Supplementary Figure S6. Hyperparameter tuning process for the SVM-RBF classifier.
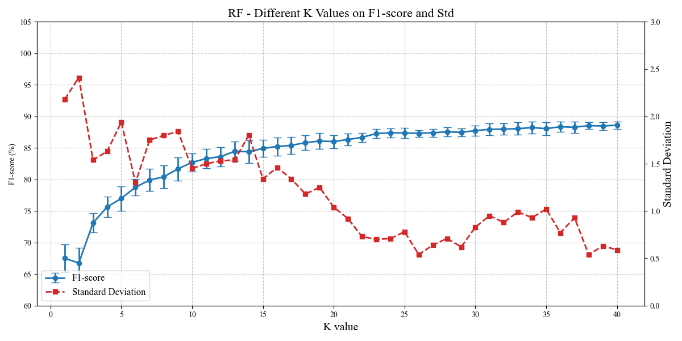


Supplementary Figure S7. Hyperparameter tuning process for the Random Forest classifier.


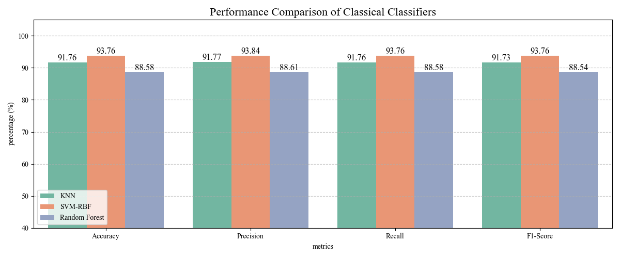


Supplementary Figure S8. Performance Comparison of Classical Classifiers

The study also highlights the importance of architectural choices in model optimization. For instance, MaxPooling was chosen for the final architecture due to its superior training stability. This is visually demonstrated by comparing the training curves: the stride = 2 model validation loss exhibits significant oscillations (Supplementary Figure S9), whereas the MaxPooling model's curve is notably smoother (Supplementary Figure S10). This observation is further supported by the lower validation loss standard deviation for MaxPooling (0.1027 vs. 0.1216) (Supplementary Table S2). Similarly, the strategic placement of a single Batch Normalization (BN) layer and the inclusion of a bottleneck layer were critical in incrementally improving the model's accuracy to its final high-performing state.


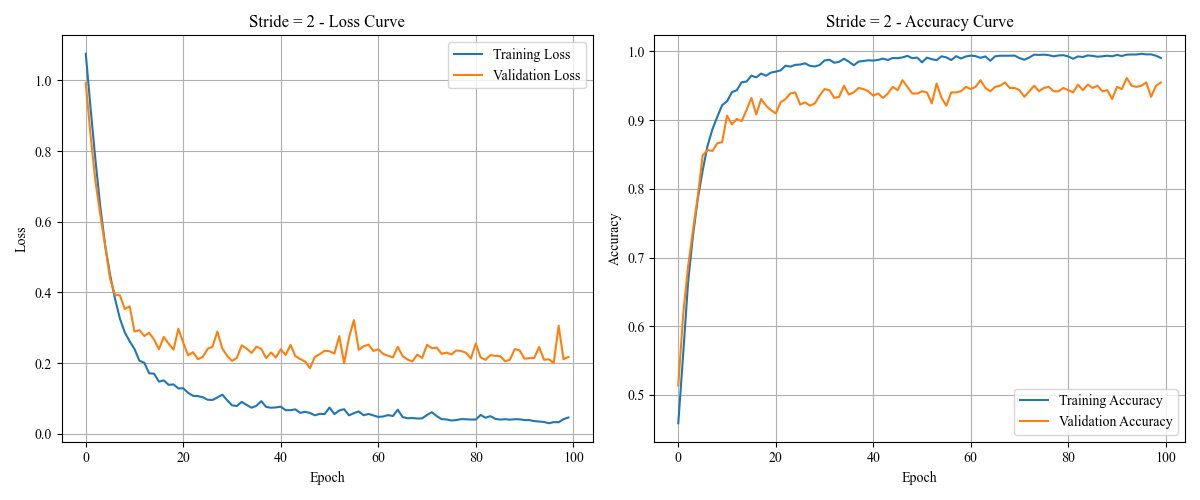


Supplementary Figure S9. Training Curves for the Encoder-augmented CNN with stride = 2.


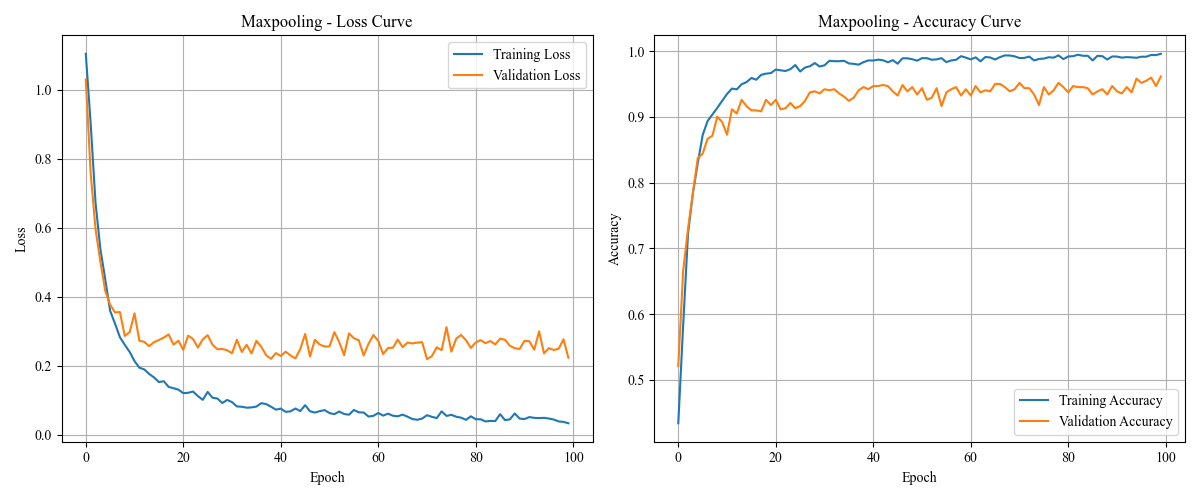


Supplementary Figure S10. Training Curves for the Encoder-augmented CNN with Max Pooling.

Supplementary Table S2. Training stability and computation time for stride and MaxPooling configurations.

| **Downsampling method** | **Validation set loss standard deviation** | **Time taken (seconds)** |
| --- | --- | --- |
| stride | 0.1216 | 1053.53 |
| MaxPooling | 0.1027 | 2989.92 |
